# A win-win solution?: A critical analysis of tiered pricing to improve access to medicines in developing countries

**DOI:** 10.1186/1744-8603-7-39

**Published:** 2011-10-12

**Authors:** Suerie Moon, Elodie Jambert, Michelle Childs, Tido von Schoen-Angerer

**Affiliations:** 1 Harvard Kennedy School and School of Public Health, Boston, USA; 2 Médecins Sans Frontières, Campaign for Access to Essential Medicines, Geneva, Switzerland

**Keywords:** Essential Medicines, Tiered Pricing, Differential Pricing, Access to Medicines, Developing Countries, Low- and Middle-income countries (LMIC), Drugs, Pharmaceuticals, Market segmentation

## Abstract

**Background:**

Tiered pricing - the concept of selling drugs and vaccines in developing countries at prices systematically lower than in industrialized countries - has received widespread support from industry, policymakers, civil society, and academics as a way to improve access to medicines for the poor. We carried out case studies based on a review of international drug price developments for antiretrovirals, artemisinin combination therapies, drug-resistant tuberculosis medicines, liposomal amphotericin B (for visceral leishmaniasis), and pneumococcal vaccines.

**Discussion:**

We found several critical shortcomings to tiered pricing: it is inferior to competition for achieving the lowest sustainable prices; it often involves arbitrary divisions between markets and/or countries, which can lead to very high prices for middle-income markets; and it leaves a disproportionate amount of decision-making power in the hands of sellers vis-à-vis consumers. In many developing countries, resources are often stretched so tight that affordability can only be approached by selling medicines at or near the cost of production. Policies that "de-link" the financing of R&D from the price of medicines merit further attention, since they can reward innovation while exploiting robust competition in production to generate the lowest sustainable prices. However, in special cases - such as when market volumes are very small or multi-source production capacity is lacking - tiered pricing may offer the only practical option to meet short-term needs for access to a product. In such cases, steps should be taken to ensure affordability and availability in the longer-term.

**Summary:**

To ensure access to medicines for populations in need, alternate strategies should be explored that harness the power of competition, avoid arbitrary market segmentation, and/or recognize government responsibilities. Competition should generally be the default option for achieving affordability, as it has proven superior to tiered pricing for reliably achieving the lowest sustainable prices.

## Background

"Access to drugs cannot depend on the decisions of private companies

but is also a government responsibility."

- WHO Commission on Intellectual Property Rights, Innovation and Public Health (WHO 2006)

The concept of selling essential medicines (drugs and vaccines) in low- and middle-income countries (LMICs) [1] at prices systematically lower than those in industrialized countries--a practice known as tiered pricing--has received widespread support from industry, policymakers, civil society, and academics as a way to improve access to these life-saving products. International tiered pricing has been proposed as an alternative to high prices when separable high- and low-to-middle-income markets exist for a medicine and when the seller exerts significant power over pricing, such as when there is limited or no competition due to patent protection, data exclusivity, or other market-entry barriers [2].

Medicines are being patented more widely in developing countries with the implementation of the World Trade Organization Agreement on Trade Related Aspects of Intellectual Property Rights (TRIPS). At the same time, the pharmaceutical market and industry is increasingly globalizing, competitive pharmaceutical producers in LMICs are emerging, unmet health needs in the developing world remain immense, and political demand for access to new products offering significant therapeutic advance is likely to grow [3]. Against this background, assessing if and how tiered pricing supports globally equitable access to medicines is critical.

Although "still very much in its infancy [4], " tiered pricing has attracted increased attention both in the pharmaceutical sector and among public actors [5-8]. The business case for tiered pricing is strong: when markets can be separated, adapting the product price to the consumer's willingness or ability to pay is a profit-maximizing strategy. At the same time, adopting such pricing can increase consumer welfare by bringing previously unaffordable products within reach. In short, tiered pricing of pharmaceuticals has received widespread support as a "win-win-win" approach to addressing access issues [9].

However, evidence and experience suggest that, in practice, tiered pricing has a number of significant drawbacks. Examining specific drug-pricing case studies, we offer here a critique of tiered pricing, organized around three key questions: (1) How can medicines be made affordable in LMICs? (2) Who should pay for research and development (R&D) and how much? (3) Who decides pricing and how?

## Discussion

### Key Concepts

#### Tiered pricing versus equity pricing

Various terms are often used synonymously with, or are related to, tiered pricing [8], including "differential pricing, " "market segmentation, " "price discrimination, " and less frequently "Ramsey pricing [2,10]." We use the term "tiered pricing" to refer to the practice of systematically setting higher prices in higher-income markets and lower prices in lower-income markets, such that there is some positive correlation between price and income. Notably, tiered pricing does not necessarily imply that a price is equitable or affordable; rather, it simply means that different prices are charged to different segments of the market for the same product.

In contrast, the concept of "equity pricing" focuses on affordability and is closely linked to the World Health Organization (WHO) concept of essential medicines, which are "intended to be available within the context of functioning health systems at all times in adequate amounts...at a price the individual and the community can afford [11]." Equity pricing emphasizes the perspective of the consumer, ie, whether a price is affordable and acceptable to him or her. In contrast, tiered pricing emphasizes the perspective of the producer, ie, whether a fair profit can be sustained while charging lower prices to lower-income populations. While tiered pricing *may *lead to equitable prices, the concepts are not equivalent and there is no guarantee that tiered prices are affordable.

#### Affordability

When is a medicine price equitable or affordable? Measuring affordability is not straightforward and depends on various factors, including the purchaser (eg, individual, household, community, private insurer, national health system, or international donor) and product specificities (eg, whether the expense is one-time or recurring).

Different approaches to measuring affordability have been proposed, including benchmarking medicine prices against per capita gross national income (GNI), setting prices against "catastrophic" household health expenditure levels [12], or converting prices to working days based on government salaries as a proxy for average income [13]. However, since these methods do not account for widely varying levels of income inequality in different countries [14], Niëns *et al. *developed a methodology to measure affordability based on the proportion of a population that would be pushed below a poverty line (either $1.25 or $2 per capita/day) by the purchase of a medicine, ie, "the impoverishing effect of a medicine." [15] This methodology offers the advantage of comparability across time and countries using available data, while being sensitive to widely varying income distributions within countries.

Regardless of the range of possible measures of affordability, actually achieving or approaching affordability, particularly for the poorest populations, likely requires attaining the *lowest sustainable price*, which approaches the cost of production in a competitive market. We define "lowest sustainable price" here as one that provides sufficient profit to the producer to incentivize ongoing production.

### Evidence from Drug Pricing Case Studies

We reviewed experiences with tiered pricing over the past decade across a diverse set of products, including antiretrovirals for HIV/AIDS, artemisinin-combination therapy for malaria, treatments for drug-resistant tuberculosis, drugs for visceral leishmaniasis, and the pneumococcal vaccine; we found that each case offered distinct insights regarding the relative utility of tiered pricing, as detailed in the following sections.

#### HIV/AIDS: Antiretrovirals

Among different therapeutic areas, the availability of data and analysis on tiered pricing is greatest for HIV/AIDS. In a review of over 7,000 developing-country purchase transactions from 2002-2007, Waning *et al. *found that the tiered prices for 15 of 18 antiretroviral (ARV) drugs were 23-498% higher than the generic price [16]. As of mid-2011, of the three products for which tiered prices were lower than generic prices, two products now have lower-cost generics available.

Similarly, an analysis of publicly announced prices found that of the 30 products for which both originator tiered prices and WHO pre-qualified generic prices were listed, the generic price was lower for 27 products (90%) [17]. In at least one case, the generic price fell between the originator's Category 1 (roughly, lower-income countries) and Category 2 (roughly, middle-income countries) tiered price.

**Figure 1:**
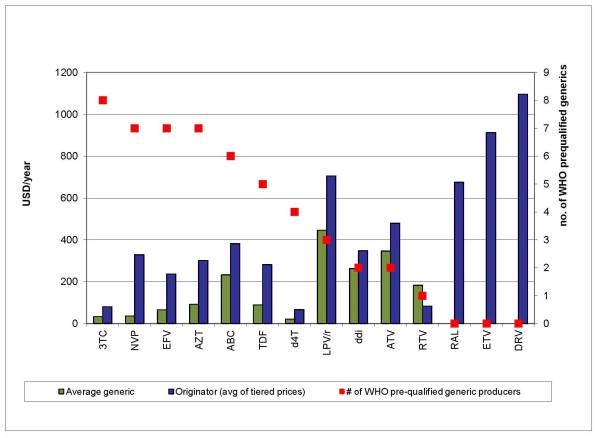
**Number of competing WHO-prequalified suppliers by antiretroviral product**. All prices are per patient/per year. 3TC = lamivudine 150 mg; NVP = nevirapine 200 mg; EFV = efavirenz 600 mg; AZT = zidovudine 300 mg; ABC = abacavir 300 mg; TDF = tenofovir 300 mg; d4T = stavudine 30 mg; LPV/r = lopinavir/ritonavir 200/50 mg; ddI = didanosine 400 mg enteric coated; ATV = atazanavir 150 mg; RTV = ritonavir 100 mg; RAL = raltegravir 400 mg; ETV = etravirine 100 mg; DRV = darunavir 300 mg. Source: MSF 2011 [17]

Finally, medicines prices tend to fall further when more competitors enter the market [18] (Figure 1), though the optimal number of competitors for a given product market will depend on a number of factors, including market size, economies of scale in production, and regulatory measures to prevent oligopolistic or collusive pricing [19,20].

Overall, the evidence from ARVs strongly suggests that generic prices are generally lower than tiered prices, and that competition among multiple producers systematically results in dynamic price reductions.

##### The special case of lopinavir/ritonavir

The price history of the fixed-dose combination (FDC) ARV lopinavir/ritonavir (LPV/r; Kaletra^®^) merits special attention, both for its complexity and the lessons it holds regarding the potential pitfalls of tiered pricing. LPV/r is a critical drug for second-line HIV/AIDS treatment; the 2009 WHO HIV/AIDS treatment guidelines recommend only one other protease inhibitor (PI), atazanavir (ATV), which must be taken together with ritonavir but is not yet available as an FDC with ritonavir [21]. Since 2006, LPV/r has been available in a heat-stable formulation that is well-suited for settings where refrigeration is scarce. LPV/r is by far the most widely used PI in developing countries, administered to 93% of adults on second-line treatment [22].

**Figure 2:**
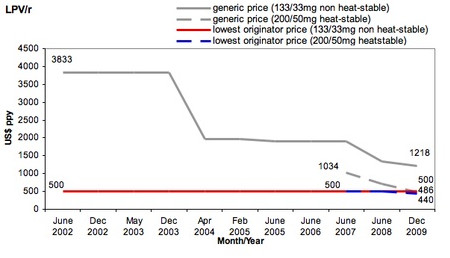
**Lopinavir/ritonavir price trends, 2002-2009**. Source: MSF 2010 [17]

Abbott Laboratories holds the patents on lopinavir and ritonavir and initially announced a tiered price of $650 for LPV/r in 2001 for African countries and 16 non-African least developed countries (LDCs) [23]. In 2002, Abbott announced a price drop to $500 for all African countries and LDCs (Category 1 countries). From 2002-2009, Abbott's price for Category 1 countries did not change (Figure 2). During this period, the lowest generic price for LPV/r remained above $500, ie, no effective price competition existed in the market. In August 2009, the Clinton HIV/AIDS Initiative (CHAI) announced that generic LPV/r would be available at $470, the first time the generic price fell below Abbott's tiered price. Several weeks later, for the first time in 7 years, Abbott reduced its price, dropping it to $440, or slightly below the lowest generic price. This history suggests that producers do not have strong incentives to reduce tiered prices in the absence of competition, nor are tiered prices immune to competition when it does arise.

While Category 1 countries received the lowest global price for LPV/r for a number of years, excluded countries negotiated prices with Abbott case by case, often resulting in very high prices. For example, in 2005 the price of LPV/r offered to Médecins Sans Frontières (MSF) programs in China was $5,000 [24], while in 2006 Honduras paid $7,775. Under strong civil society pressure, in 2006 Abbott offered a Category 2 tiered price of $2,200 for a group of 40 developing countries excluded from its initial offer. However, some governments considered this price too high, and after Thailand issued a compulsory license on the drug in January 2007, Abbott dropped the Category 2 price again by more than half to $1,000. As of July 2010, the price has remained at $1,000 for a group of 45 LMICs. The pricing of LPV/r for non-Category 1 countries illustrates the difficulty of setting equitable and affordable tiered prices across diverse country contexts.

Finally, tiered pricing may have anti-competitive effects if the price is so low that it discourages market entry by potential competitors. Abbott controlled 80-100% (by volume) of the developing-country LPV/r market from 2006 to 2008 [25]. Abbott's dominance in this market contrasts with other ARVs, in which generics supply 80% or more of the developing-country market (by volume). Questions have been raised regarding whether the company's pricing policies were intended to prevent competition [26-28]. While consumers may benefit in the short term from tiered prices set below production costs, the resultant lack of competition and absence of dynamic price reductions means that consumers may pay higher prices in the long term.

#### Malaria: Artemisinin-based Combination Therapies

As with ARVs, evidence from the market for artemisinin-based combination therapy (ACT) drugs for malaria shows that generic competition yields lower prices than tiered pricing alone.

**Figure 3:**
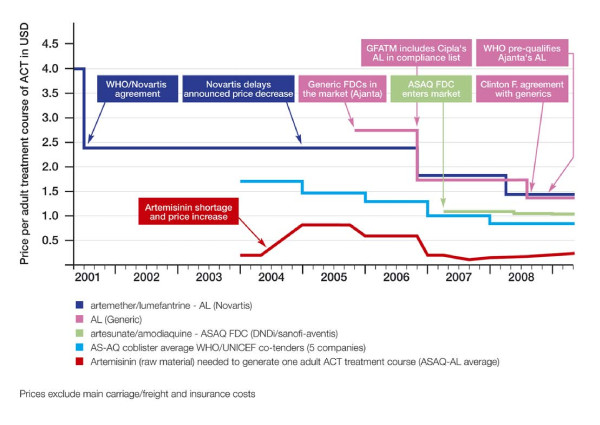
**Artemisinin-based combination therapy drug price trends 2001-2008**. Source: Moon *et al. *2009 [30]

In 2001, Novartis offered WHO an "at-cost" tiered price for developing countries of $2.40 per adult treatment course for artemether-lumefantrine (AL; Coartem^®^) [29]. For several years, AL was the only fixed-dose combination ACT that met the quality requirements of the WHO or Global Fund to Fight AIDS, Tuberculosis and Malaria (GFATM); therefore, no competition existed in the donor-funded ACT market. The tiered price did not change for 5 years (Figure 3) [30]. After a generic version of AL became eligible for GFATM purchase, Novartis decreased its price to $1.80, then dropped its tiered price again to $1.50 shortly after the FDC of artesunate-amodiaquine (ASAQ), a substitute and competitor to AL in some countries, entered the market at $1.00.

Evidence from the global ACT market suggests that competition helps reduce tiered prices, and underscores the need to ensure that "at-cost" pricing is independently verifiable and reflects changes in production costs over time.

#### Tuberculosis: Medicines for Drug-Resistant Disease

Most medicines for tuberculosis (TB) are widely available as low-cost generics, but some medicines for drug-resistant TB (DR-TB, including multidrug-resistant and extensively drug-resistant TB) can be quite costly, and may be offered at a tiered price. Eli Lilly produced two key DR-TB drugs, capreomycin and cycloserine, which are off patent but not widely available from other suppliers. In 2002, Lilly began supplying the drugs at a "preferential price" through the WHO Green Light Committee (GLC), and transferred the technology to produce the drugs and its active pharmaceutical ingredient (API) to several generic drug companies in TB-endemic countries. Unlike for ARVs or ACTs, the tiered price has consistently remained below the generic prices for these drugs.

Capreomycin: As of September 2011, no generic sources of capreomycin were WHO Pre-Qualified (PQ). In 2001, GLC-approved programmes were able to access Lilly's capreomycin for $1.02 per vial. Since 2001, the price has increased nearly four-fold to $4.00 per vial for the WHO Global Drug Facility [31]. A further price increase is expected in the near future, now that Lilly has stopped production for this market, and the GDF begins sourcing capreomycin from the pharmaceutical producer Akorn, which has reported a price of $8 per vial (Table 1) [31]. While the Akorn price to the GDF is still relatively high, it is lower than the price found in industrialized countries of $40.95 per vial [32].

**Table 1:** Capreomycin tiered prices

Manufacturers	Akorn	Mac Leods	GDF pooled procurement price
**Quality status**	Approved by a Stringent Regulatory Authority	Under evaluation by WHO PQ	GDF Quality Assurance Policy

**1 g powder for injection**	8.00	No price information given	4.00* (Eli Lilly)

Price and quality information. Price of the lowest unit (i.e. the price of one tablet, capsule or vial) in USD ($)

* In future, the lowest available price is expected to change as the Akorn product replaces Eli Lilly's.

Sources: British National Formulary 2010 [32]; Global Fund 2010 [54]; Management Sciences for Health 2009 [55]; personal communication, M. Price, 2010.

Cycloserine: By 2009, after technology transfer from Lilly, two generic WHO prequalified sources of cycloserine were available. In 2001, GLC-approved programmes were able to access Lilly's cycloserine for $0.14 per capsule, but in 2008 Lilly stopped producing the drug. The price of cycloserine has since increased by over four-fold to $0.59 per capsule (Table 2) [31]. However this price remains considerably more affordable than prices paid in high-income countries; for example, the British National Formulary lists a price of $5.43 per capsule [32].

**Table 2:** Cycloserine tiered prices

Manufacturers	Lupin	Aspen	Mac Leods	Purdue GMP	GDF pooled procurement price
**Quality status**	Under evaluation by WHO PQ	Approved by WHO PQ	Approved by WHO PQ	Approved by a Stringent Regulatory Authority	GDF Quality Assurance Policy

**250 mg capsule**	0.60	0.78	No price information given	No price information given	0.59 and 0.78 (Macleods and Aspen)

Price and quality information. Price of the lowest unit (i.e. the price of one tablet, capsule or vial) in USD ($)

Sources: British National Formulary 2010 [32]; Global Fund 2010 [54]; Management Sciences for Health 2009 [56]; personal communication, M. Price, 2010.

This experience suggests that under special circumstances tiered pricing may result in lower prices than competitive production. When demand is low and production capacity limited, a single producer selling at tiered prices in developing countries may result in lower prices than would otherwise be feasible. Notably, the GLC has approved a *cumulative *total of only 49,858 patient treatments from 2000-2008 [33]. In 2009, only 30,475 of the estimated 440,000 new patients with MDR TB were started on treatment [34]. While the market for DR TB drugs is growing, the patient numbers remain quite small. Generic prices may fall as global volumes increase, producers achieve economies of scale, and/or more producers of API or finished products enter the market. Nevertheless, from 2000 when Lilly began supplying capreomycin and cycloserine to the GLC, until generic suppliers were able to takeover supply (2007 for cycloserine, projected 2011 or later for capreomycin), Lilly's tiered price has likely helped to ease access problems related to the cost of DR-TB drugs.

#### Visceral Leishmaniasis: Liposomal Amphotericin B

Amphotericin B is used to treat fungal infections, as well as visceral leishmaniasis (VL; kala azar), a fatal neglected tropical disease highly endemic in India, Bangladesh, Nepal, Sudan, Ethiopia, and Brazil. Amphotericin B is better tolerated by patients who do not respond well to sodium stibogluconate, the standard VL treatment in some countries, and is recommended for treatment of VL patients co-infected with HIV. Use of the liposomal formulation of amphotericin B (AmBisome^®^, produced by Gilead Sciences) has significantly fewer side effects than conventional amphotericin B [35].

In 1992, Gilead agreed with WHO to supply liposomal amphotericin B (LAmB) for treatment of VL to developing countries at cost plus 10%, ie, $50/vial ($700/treatment) (Table 3). In 2005, an informal WHO expert consultation recommended using LAmB to treat VL and highlighted the need for wider access. The following year, Gilead and WHO agreed on a tiered price of $20/vial for VL and mucosal leishmaniasis in developing countries; in August 2009, Gilead reduced the price further to $18/vial and committed to update the price annually depending on its production cost, with a price ceiling of $20 (personal communication, G. Alton, 2009). In India, Cipla marketed a generic LAmB version for $140/vial and offered a specially discounted price for VL treatment of $25/vial. The UK private sector price in 2010 was $153/vial.

**Table 3:** Liposomal amphotericin B tiered prices (prices in USD ($)

	Average unit price (year)*		Average treatment cost (year)	
**Liposomal amphotericin B**	**Gilead**	**Lowest generic (Cipla VL price)**	**Gilead**	**Lowest generic (Cipla VL price)**

**WHO**	18 (2009)	25 (2008)	252 (2009)	350 (2008)
	
	20 (2006)		280 (2006)	
	
	50 (1991)		700^† ^(1991)	

**UK private market**	153 (2010)		2,142 (2010)	

*Currency converted using http://www.oanda.com on 13 December 2010.

^† ^Based on an average weight of 35 kg for a patient with visceral leishmaniasis, 14 vials are required for a full treatment course.

VL = visceral leishmaniasis.

Sources: personal communication, G. Alton, 2009.

The case of LAmB suggests that it is possible to segment markets by indication. However, similar discounts are also needed for use against other fungal infections such as meningococcal meningitis in people living with HIV/AIDS, illustrating the public health limits of segmenting markets by indication. With only two producers, the market for this formulation is not yet competitive; but in the medium term, if the market expands and can attract more competitors, prices for LAmB may decrease and make this drug more accessible for both patients with VL and those with other fungal infections. In the short term, the tiered prices most likely increased access to this medicine specifically for the treatment of VL.

#### New Vaccines: Pneumococcal Vaccine

Older vaccines have long been available at relatively low cost in developing countries. However, newer, more expensive vaccines have been recently developed, such as for rotavirus, human papillomavirus, and pneumonia, raising questions regarding access in developing countries. In 2010, controversy arose around the tiered pricing of pneumococcal vaccines.

For three decades, the Pan American Health Organization (PAHO) has procured vaccines at low prices for Latin America through its Revolving Fund; by aggregating demand across a set of small- and medium-sized countries, the Revolving Fund strengthened the negotiating leverage of governments vis-à-vis suppliers. Revolving Fund contracts include a "most favored nation" clause that requires suppliers to give PAHO their lowest available price. However, with most Latin American countries falling into the lower-middle or upper-middle income categories, the requirement that PAHO receive the lowest global prices has clashed with producers' tiered pricing strategies, which charge higher prices to middle-income countries.

In 2008-2009, PAHO negotiated a price of $21.75/dose for Wyeth's pneumococcal 7-valent conjugate vaccine (Prevnar^®^). A 10-valent vaccine protecting against a broader range of serotypes was developed by GSK (Synflorix^®^), which will supply it through the Advance Market Commitment (AMC) mechanism of the Global Alliance for Vaccines and Immunization (GAVI) for up to 72 developing countries [36]. The initial price is $7/dose for approximately 20% of the total quantity provided, which then decreases to a "tail" price of $3.50/dose for the remainder [37]. PAHO could not obtain the same price from GSK and initially decided not to purchase the 10-valent vaccine.

**Figure 4:**
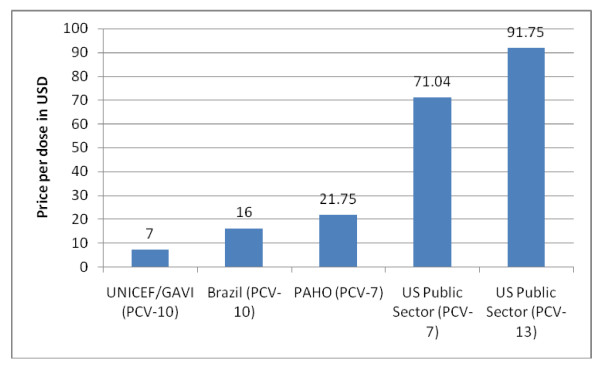
**Pneumococcal conjugate vaccine tiered prices**.

In parallel, the government of Brazil negotiated an 8-year agreement with GSK to purchase the 10-valent vaccine initially at $16/dose (Figure 4), decreasing to $7 in later years; the beginning and tail prices are roughly double the GAVI price. GSK has also agreed to transfer technology to the Brazilian public manufacturer BioManguinhos to produce the vaccine by the end of the 8-year period. The same vaccine is sold at $49-56/dose in Europe and $71/dose in the US [38]. The Brazil-GSK agreement may have had a price setting effect for the region as PAHO subsequently accepted a price of $14.85/dose. GSK furthermore succeeded in separating the GAVI and PAHO markets with two different presentations of the same vaccine (two and one dose vials respectively).

The pneumococcal vaccine case highlights several difficulties with tiered pricing. First, no mechanism is in place ensuring that poorer countries get the lowest possible prices. Under the AMC/GAVI agreement, the price of the pneumococcal vaccine is fixed at $7, then $3.50 for the next 10 years, but the production cost has been estimated at $1-3/dose [39], suggesting that prices would have fallen further over time in a competitive market. These price differences may be particularly important for LMICs that will become ineligible for GAVI funding in the coming years, and will therefore need to pay for vaccines from national budgets. Second, this case underscores the difficulty in determining what is a "fair" price for middle-income countries. Finally, the Brazil-GSK deal suggests that, while large countries with domestic manufacturing capacity may be able to negotiate acceptable prices and technology transfer agreements, for smaller countries without production capacity, the practice of negotiating prices country by country may be less favorable.

In summary, when synthesizing the past decade's experience with tiered pricing in HIV/AIDS, malaria, tuberculosis, visceral leishmaniasis and pneumococcal vaccines, we found that when markets were sizeable and multiple sources of production were available, tiered pricing performed poorly compared to competitive production in generating reliable and sustained price reductions. We also found that in special cases, particularly when markets were small, highly uncertain, where production capacity was limited, or there was a time delay to overcoming barriers to competition, tiered pricing likely contributed to improved access in the short term. However, beyond price, we also found that tiered pricing raised further issues requiring consideration as discussed in the following sections.

### Tiering and Pricing: Variations vs Principles

#### Differential and Arbitrary Tiering Among Countries

Current tiered pricing policies take various approaches to country classification. Drug companies rarely provide an explicit rationale for why they offer their lowest prices to some countries, somewhat higher prices for others, and for still others negotiate prices on a case-by-case basis. Some companies use World Bank income classifications based on per capita GNI (low, lower-middle, upper-middle, and high-income), while others use development indicators, such as the UN-designated LDCs or the UN Development Programme's Human Development Index. Finally, some companies offer their lowest prices, such as for ARVs, to all sub-Saharan African countries, regardless of income or LDC status, presumably because of the disproportionately high burden of HIV in the region.

For example, for ARV pricing, Bristol-Myers Squibb includes 57 developing countries in its Category 1, primarily low-income and African countries, but places southern African countries in Category 2. Southern Africa, however, has the highest HIV-prevalence rates in the world. The impact of this categorization is that Bristol-Myers Squibb prices its important second-line drug atazanavir 25% higher at $547 in southern Africa, compared with $412 in other countries where HIV prevalence is lower and, in a few cases, income is higher [17].

Thus, tiered pricing policies are not necessarily logical nor correlated with need or ability to pay, though that is the purported objective. How companies decide to put countries into different pricing tiers is not always clear, nor is there consensus on the criteria by which to do so.

#### Intra-Country Price Differences

##### Within-country market segmentation

In some cases, within-country tiered pricing has been proposed and/or implemented, and evidence suggests internal market segmentation may be feasible. For example, Yadav has presented evidence from the malaria drug market in sub-Saharan Africa suggesting that price differences can be maintained between "premium" and "non-premium" private sector distribution channels, through branding and other marketing strategies [40]. However, the distributional effects of particular segmentation policies should also be examined.

Perhaps the most common approach is to segment the public and private sectors, with lower prices for government-provided medicines. However, such a simple division may not be equitable, since countries vary widely in the extent to which a population purchases medicines in the public or private sector, and private sector customers are not necessarily wealthier than those who rely on the public sector. For example, over 70% of the TB drug market in India and the Philippines is in the private sector, while in Brazil and South Africa, TB drugs are mostly dispensed via the public sector [41].

In addition, in a study of 36 countries Cameron *et al. *found the availability of medicines to be higher in the private than public sector, though public sector prices tended to be lower [42]. Although incomes are generally lower in rural areas, public health centers are often sparse, meaning that poor, rural populations may rely on private dispensaries to purchase medicines. Thus, a tiered pricing model that only offers affordable prices to the public sector could exclude a substantial proportion of the poor population in some countries.

Another proposed method to achieve internal market segmentation is to charge higher prices in the insured market, while offering lower prices for all other sectors, including public, private, and non-profit (personal communication, K. Outterson, 2010). If such a policy could feasibly be implemented, and if health insurance coverage were sufficient to pay for the prices of needed medicines, then such a division could lead to more equitable distributional outcomes than a simple public-private sector division. However, many developing countries having extremely limited insurance systems with very low coverage.

##### Within-country inequality

A key weakness with pricing medicines according to per capita GNI levels is that many middle-income countries are also characterized by high levels of inequality. South Africa and Brazil, for example, are the 8^th ^and 10^th ^most unequal out of 182 ranked countries in the world (Table 4) [43]. Tiered prices may be within reach for the upper or middle classes in a country, but not for the poor. In a 2002 study of 13 countries, Wong found that medicine prices were higher in countries with higher levels of inequality, but per capita gross domestic product (GDP) had no significant effect on price [44].

**Table 4:** Selection of intra-country inequality scores

Country	Gini coefficient	Rank	Income category
Namibia	74.3	1^st^, most unequal	Upper middle
South Africa	57.8	8^th^	Upper middle
Brazil	57.0	10^th^	Upper middle
China	46.9	58^th^	Lower middle
East Timor	39.5	71^st^, median	Lower middle/LDC
India	36.8	87^th^	Lower middle
Bangladesh	33.4	124^th^	Low income/LDC
Denmark	24.7	182^nd^, most equal	High income

LDC = least developed country

Source: UNDP 2009 [43]

#### Setting Fair Prices: How and Who

With respect to tiered prices for middle-income countries, there is no norm for what constitutes a "fair" premium on LDC or low-income country prices. In practice, prices may be determined by many factors besides the ability to pay, such as negotiating capacity, market size, and degree of competition.

For example, in 2006, Honduras purchased LPV/r at a price about 6 times that of Brazil, although the two countries' adult HIV prevalence rates are roughly equivalent (~0.5%) and Honduras' per capita GNI is only one-fourth that of Brazil's. Brazil's larger market and ability to credibly threaten the use of compulsory licensing were likely to have contributed to the lower prices achieved there [45].

In most high-income markets, governments play a central role in regulating medicine prices, such as through reference pricing, setting reimbursement rates, and price controls. In contrast, smaller countries and those without domestic pharmaceutical industries have much less bargaining power and often face greater difficulty achieving affordable prices in case-by-case price negotiations with companies. Under tiered pricing policies, firms generally set the price and choose which countries will receive which price. In short, tiered pricing policies give most of the decision-making power to private firms, whose pricing decisions may not necessarily be aligned with the public interest.

Besides ability to pay, a range of factors could facilitate fair price setting, including therapeutic or public health value, drug production costs, total R&D costs, and public investment in R&D. Lopert *et al. *proposed setting tiered prices based on pharmacoeconomic principles [46]: setting a fair, objectively calculated price taking into account a product's public health benefit, cost-benefit ratio, availability of alternatives, potential cost savings in other parts of the healthcare system, and degree of public or philanthropic R&D funding. Such a system could shift the alignment of rewards for innovation closer to public health needs, rather than market profitability. To our knowledge, this approach has not yet been applied in developing countries, but such systems are in place in the UK, Canada, and Australia, where national governments are major purchasers of pharmaceuticals. However, pharmacoeconomic approaches may need to be combined with other measures to achieve equity pricing of important medicines [47].

## Summary

Contrary to the idea that tiered pricing is a "win-win" solution, this review of the evidence and literature suggests key economic and political drawbacks to this policy tool. Currently, there is no straightforward, equitable way to set tiered prices to achieve affordability.

First, tiered pricing does not necessarily result in the lowest sustainable prices, nor does it reliably lead to price reductions over time. In comparison, when markets are sufficiently large and multiple sources of production exist, robust competition has consistently proven across different therapeutic areas to result in lower prices. Second, no clear international norm has been established for setting price tiers, nor is there a simple or satisfactory way to allocate payment for R&D costs across various developing countries. The distributional nature of the question is fundamentally political rather than technical. Finally, tiered pricing policies give too little decision-making power to governments, which are accountable to their populations under international law for ensuring access to medicines. Rather, tiered pricing leaves this important issue almost entirely in the hands of private companies over which populations have few means to demand accountability.

In special cases however, such as when market volumes are very small or highly uncertain (eg, drug-resistant TB) and/or multisource production capacity is lacking (eg, newer products like the pneumococcal vaccine), tiered pricing may offer the only practical *short-term *option to increase access to a product. In such cases, tiered pricing should be implemented in the short term, while simultaneous steps are taken to improve affordability and availability over the longer term. Where markets are small and economies of scale make a single producer the most efficient solution, credible means to verify "at-cost" or "cost-plus" supply commitments are needed. In cases where global production capacity is limited, policies should encourage rapid technology transfer to transition as quickly as possible to a competitive market. In general, competition should be the default option for improving the affordability of medicines in developing countries. Increasingly, ensuring such competition will require addressing the challenges posed by more widespread patenting of medicines in developing countries [48]. These conclusions have particular policy relevance for major purchasers of medicines, who have the power to shape markets for these products; such actors include governments of large developing countries and global health initiatives such as GAVI, the Global Fund (which has a Market Dynamics Committee), UNITAID (which is centrally-focused on market dynamics), and the United Nations Childrens Fund (UNICEF).

If not tiered pricing, then what? Given the many difficulties around setting tiered prices, pricing through competition offers clear advantages. Making competitively produced medicines available in all developing countries would minimize the complications of internal market segmentation, since the lowest prices would be available across all sectors. Policies enabling such competition merit further attention. Such policies may include voluntary measures by patent holders such as widespread voluntary licensing, participating in the UNITAID-supported Medicines Patent Pool [49], non-assert declarations, or decisions not to apply for or maintain patents in developing countries. They may also include policies adopted by governments, such as regular compulsory licensing where patents exist, or limiting patent grants through strict patentability criteria and procedures to facilitate pre- and post-grant oppositions on patent applications.

However, such a system will only work in the long term if markets are large enough and alternate solutions for financing R&D can be implemented. The current system relies on the ability of producers to recoup R&D investments by charging a significant market premium above production costs. If alternate models could be implemented that "de-link" medicine prices from R&D costs, many of the thorny economic, logistical, and political problems raised by tiered pricing could be averted. Proposals that de-link prices from R&D include push funding, prizes [50,51], patent pools [52], and patent buy-outs [53]. Furthermore, R&D policies could also incentivize developers to take production costs into account throughout the development process, making affordability of the end product more feasible. Some public-private product development partnerships (PDPs) targeting resource-poor settings already consider production costs when deciding which compounds to pursue.

A political process is required to determine how countries should contribute to R&D financing as a global public good. The debate on this issue advanced through the 2-year WHO Intergovernmental Working Group on Public Health, Innovation and Intellectual Property (IGWG, 2006-2008) process, which resulted in the Global Strategy and Plan of Action on Public Health, Innovation and Intellectual Property (GSPoA). It is too early to draw conclusions, as implementation of the GSPoA is just beginning and work continues on key questions regarding R&D financing. Nevertheless, the international community is clearly seeking policy solutions that extend beyond the limited benefits of tiered pricing, in order to institute systemic change that will improve access to medicines for all.

## Competing interests

The authors declare that they have no competing interests.

## Authors' contributions

SM drafted the manuscript and participated in conception, design, and analysis. EJ, MC, and TvSA participated in conception, design, analysis, and interpretation, and revised critically the manuscript. All authors read and approved the final manuscript.
